# Ego-Dissolution and Psychedelics: Validation of the Ego-Dissolution Inventory (EDI)

**DOI:** 10.3389/fnhum.2016.00269

**Published:** 2016-06-14

**Authors:** Matthew M. Nour, Lisa Evans, David Nutt, Robin L. Carhart-Harris

**Affiliations:** ^1^Psychiatric Imaging Group, MRC Clinical Sciences Centre, Imperial College LondonLondon, UK; ^2^Institute of Psychiatry Psychology and Neuroscience, King’s College LondonLondon, UK; ^3^Faculty of Medicine, Centre for Neuropsychopharmacology, Division of Brain Sciences, Imperial College LondonLondon, UK

**Keywords:** ego dissolution, ego disintegration, ego death, psychedelic, self disturbance, ego boundaries, mystical experience, hallucinogen

## Abstract

**Aims**: The experience of a compromised sense of “self”, termed ego-dissolution, is a key feature of the psychedelic experience. This study aimed to validate the Ego-Dissolution Inventory (EDI), a new 8-item self-report scale designed to measure ego-dissolution. Additionally, we aimed to investigate the specificity of the relationship between psychedelics and ego-dissolution.

**Method**: Sixteen items relating to altered ego-consciousness were included in an internet questionnaire; eight relating to the experience of ego-dissolution (comprising the EDI), and eight relating to the antithetical experience of increased self-assuredness, termed ego-inflation. Items were rated using a visual analog scale. Participants answered the questionnaire for experiences with classical psychedelic drugs, cocaine and/or alcohol. They also answered the seven questions from the Mystical Experiences Questionnaire (MEQ) relating to the experience of unity with one’s surroundings.

**Results**: Six hundred and ninety-one participants completed the questionnaire, providing data for 1828 drug experiences (1043 psychedelics, 377 cocaine, 408 alcohol). Exploratory factor analysis demonstrated that the eight EDI items loaded exclusively onto a single common factor, which was orthogonal to a second factor comprised of the items relating to ego-inflation (rho = −0.110), demonstrating discriminant validity. The EDI correlated strongly with the MEQ-derived measure of unitive experience (rho = 0.735), demonstrating convergent validity. EDI internal consistency was excellent (Cronbach’s alpha 0.93). Three analyses confirmed the specificity of ego-dissolution for experiences occasioned by psychedelic drugs. Firstly, EDI score correlated with drug-dose for psychedelic drugs (rho = 0.371), but not for cocaine (rho = 0.115) or alcohol (rho = −0.055). Secondly, the linear regression line relating the subjective intensity of the experience to ego-dissolution was significantly steeper for psychedelics (unstandardized regression coefficient = 0.701) compared with cocaine (0.135) or alcohol (0.144). Ego-inflation, by contrast, was specifically associated with cocaine experiences. Finally, a binary Support Vector Machine classifier identified experiences occasioned by psychedelic drugs vs. cocaine or alcohol with over 85% accuracy using ratings of ego-dissolution and ego-inflation alone.

**Conclusion**: Our results demonstrate the psychometric structure, internal consistency and construct validity of the EDI. Moreover, we demonstrate the close relationship between ego-dissolution and the psychedelic experience. The EDI will facilitate the study of the neuronal correlates of ego-dissolution, which is relevant for psychedelic-assisted psychotherapy and our understanding of psychosis.

## Introduction

Distortions in the subjective experience of one’s “self”, or “ego”, are central to the psychedelic experience (James, 1882; Huxley, 1954; Savage, 1955; Klee, 1963; Leary et al., 1964; Grof, 1976, 1980; Harrison, 2010; Carhart-Harris et al., 2014; Lebedev et al., 2015). Specifically, a reduction in the self-referential awareness that defines normal waking consciousness has been reported with all classical psychedelic drugs (5-HT_2A_ receptor agonists), including psilocybin (Griffiths et al., 2008, 2011), lysergic acid diethylamide (LSD; Goodman, 2002; Lyvers and Meester, 2012), and dimethyltryptamine (DMT; Trichter et al., 2009), as well as with other psychoactive substances such as nitrous oxide (James, 1882) and ketamine (Vollenweider and Kometer, 2010).

The experience of a compromised sense of self occasioned by psychedelic drugs has been variously called ego-death (Grof, 1980; Harrison, 2010), ego-loss (Leary et al., 1964), ego-disintegration (Muthukumaraswamy et al., 2013; Lebedev et al., 2015) and ego-dissolution (Klee, 1963; Studerus et al., 2010; Carhart-Harris et al., 2014; Lebedev et al., 2015; Tagliazucchi et al., 2016). This experience has been interpreted from a psychoanalytic perspective as a disruption of ego-boundaries, which results in a blurring of the distinction between self-representation and object-representation, and precludes the synthesis of self-representations into a coherent whole (Federn, 1952; Savage, 1955; Fischman, 1983).

It is likely that the prior “psychology” of the subject and the environmental setting in which they take a psychedelic influences whether an ego-dissolution experience is welcomed and felt as something positive, or feared and fought against (Eveloff, 1968; Fischman, 1983; Griffiths et al., 2008; Studerus et al., 2010, 2012). At one extreme, ego-dissolution is closely related to blissful mystical experiences such as can be occasioned by certain spiritual or religious practices (Stace, 1960; Hood, 1975; MacLean et al., 2012); indeed, loss-of-self was identified by William James as being a cardinal feature of the mystical experience (James, 1985). These experiences are characterized by a feeling of unity with one’s surroundings, which is explicitly related to disturbed ego-boundaries and thus ego-dissolution. Moreover, the mystical experience is likely to be of therapeutic benefit in psychedelic-assisted psychotherapy (Leary et al., 1964; Grof, 1980; Griffiths et al., 2008, 2011; Johnson et al., 2008, 2014). At the other extreme, it has been argued that self-disturbances and disturbed ego-boundaries are a core phenomenological feature of psychosis and schizophrenia (Bleuler, 1950; Laing, 1959; Scharfetter, 1981; Fischman, 1983; Parnas, 2011; Sass et al., 2011; Northoff, 2014; Nour and Barrera, 2015), although it remains unclear precisely how the self-disturbances specific to schizophrenia relate to the experience of ego-dissolution under psychedelics.

Discussions of altered self-experience have been traditionally confined to philosophy or descriptive psychopathology (Stace, 1960; Jaspers, 1997). In recent years, however, there has been increased interest in the neurobiological correlates of the experience of self (Carhart-Harris and Friston, 2010; Qin and Northoff, 2011; Carhart-Harris et al., 2014). Psychedelic drugs may provide a fruitful avenue of research into the neuronal correlates of normal and abnormal self-awareness or ego-consciousness (Carhart-Harris et al., 2013, 2014; Muthukumaraswamy et al., 2013; Roseman et al., 2014; Lebedev et al., 2015; Tagliazucchi et al., 2016). This research programme, however, is predicated on the existence of a validated self-report measure of the ego-dissolution experience.

There currently exist several measures that capture feelings related to alterations in self-experience (Strassman et al., 1994; Parnas et al., 2005; Studerus et al., 2010; MacLean et al., 2012). Dittrich’s APZ (Abnormal Mental States) questionnaire, and its revised versions, OAV and 5D-ASC, have been used extensively to characterize altered states of consciousness occasioned by psychedelic drugs (Dittrich, 1998; Studerus et al., 2010). These questionnaires purport to capture both positive and negative experiences of depersonalization and derealization (“oceanic boundlessness” and “dread of ego dissolution”, respectively), as well as additional dimensions of “visionary restructuralization” (in APZ, OAV and 5D-ASC), “auditory alterations” and “vigilance reductions” (both in 5D-ASC only; Dittrich, 1998; Hasler et al., 2004; Wittmann et al., 2007; Studerus et al., 2010, 2011; Schmid et al., 2015). Recent psychometric evaluation of the OAV questionnaire, however, reveals a more complex 11-factor structure, including factors relating to changes in cognition, perception and mood, as well as to feelings of unity and disembodiment. At present, however, there are no validated scales that allow an easy, reliable and direct uni-dimensional measurement of ego-dissolution. This presents a barrier to this promising line of research.

The primary aim of the present study was to develop and validate the “Ego-Dissolution Inventory” (EDI), a new succinct 8-item self-completed questionnaire designed to operationalize the experience of ego-dissolution so that its construct validity can be tested and developed. In order to do this in an efficient way we chose to utilize online data collection via a large anonymous internet survey. A secondary aim was to investigate the specificity of the relationship between the experience of ego-dissolution and psychedelic drugs, compared with cocaine and alcohol. These two comparator drugs were chosen because of their widespread availability and use in Western societies. Finally, we aimed to test the hypothesis that experiences occasioned by classical stimulant drugs, like cocaine, are in some respects antithetical to the psychedelic experience, promoting ego-inflation rather than ego-dissolution.

## Materials and Methods

### Survey Construction

#### Ego-Dissolution Inventory Item Selection

Sixteen novel statements relating to the experience of ego-consciousness were included in this study. Eight of these were designed to capture the central phenomenon of ego-dissolution (and the associated feeling of increased union with one’s surroundings, known as dissolved ego-boundaries), and particularly how it has been characterized in the context of the psychedelic experience (Leary et al., 1964; Grof, 1980; Harrison, 2010). As well as referring to the existing literature on the psychedelic experience, we also sought the opinion of six scientists working within the field of psychedelic neuroscience when choosing the final eight ego-dissolution items, and sought consensus about the chosen items. The other eight items were designed to reflect the distinct and largely antithetical experience of unusually elevated self-assuredness and confidence (which we refer to as “ego-inflation”). The items from these two subscales were included in the final survey in an interleaved manner with the intention of minimizing question “order” effects, and the potential tendency for subjects to indiscriminately endorse any statement about altered consciousness when reflecting on an experience with a psychoactive substance (particularly if that substance can have profound and variegated psychological effects, as is the case with psychedelics). Items were rated using a visual analog scale format (0–100, with incremental units of one) with zero defined as “No, not more than usually”, and 100 defined as “Yes, entirely or completely”, taking inspiration from a previous questionnaire on altered states of consciousness developed by Dittrich (1998) as well as other self-constructed scales used internally by our team (Carhart-Harris et al., 2012, 2015; Muthukumaraswamy et al., 2013). For the exact wording of the 16 ego-consciousness items included in the survey see Table 1.

**Table 1:** **Factor loadings from exploratory factor analysis of item scores from 1828 drug experiences (extraction method: principal axis factor; rotation method: promax with Kaiser normalization; loadings taken from pattern matrix)**.

Item:	Factor 1 Ego-dissolution	Factor 2 Ego-inflation
I felt especially assertive	0.014	**0.691**
**I experienced a dissolution of my “self” or ego**	**0.883**	−0.040
I felt more important or special than others	−0.034	**0.715**
**I felt at one with the universe**	**0.830**	0.010
My ego felt inflated	−0.154	**0.756**
**I felt a sense of union with others**	**0.700**	0.167
I felt especially sure-of-myself	0.151	**0.824**
**I experienced a decrease in my sense of self-importance**	**0.663**	−0.066
I felt especially keen and competitive	−0.134	**0.725**
**I experienced a disintegration of my “self” or ego**	**0.897**	−0.070
I felt like my viewpoint was worth more than other peoples’	−0.075	**0.667**
**I felt far less absorbed by my own issues and concerns**	**0.624**	0.112
I felt especially self-confident	0.073	**0.847**
**I lost all sense of ego**	**0.885**	−0.043
I felt especially self-assured	0.156	**0.822**
**All notion of self and identity dissolved away**	**0.845**	−0.042

*Items in bold comprise the Ego-Dissolution Inventory (EDI), whilst the remaining items relate to ego-inflation. All factor loadings >0.2 are in bold*.

#### Survey Structure

Each subject was asked to provide information on their age, sex and educational background****. Subjects also provided information on their lifetime use of psychedelic drugs and cocaine, as well as their weekly alcohol consumption (all possible answer options for educational attainment and drug and alcohol use are presented in Table 2).

**Table 2:** **Demographic data for subjects who provided information for at least one drug experience**.

**Total**	691

**Female**	238 (34.44%)

**Age at time of survey**
Median	28
Inter-quartile range	13
Skewness	1.43

**Education**
Left school before age 16 (no qualifications)	2 (0.29%)
Left school at 16/GCSE (UK)	29 (4.20%)
High school diploma/A-Level (UK)	68 (9.84%)
Some university (or equivalent)	168 (24.31%)
Bachelor’s degree (or equivalent)	229 (33.14%)
Post-graduate degree (or equivalent)	195 (28.22%)

**Lifetime illicit drug use**	**Psychedelic**	**Cocaine**
Never	107 (15.48%)	236 (34.15%)
Once only	26 (3.76%)	70 (10.13%)
2–5 times	106 (15.34%)	106 (15.34%)
6–10 times	92 (13.31%)	59 (8.54%)
11–15 times	69 (9.99%)	35 (5.07%)
16–25 times	69 (9.99%)	45 (6.51%)
26–50 times	87 (12.59%)	42 (6.08%)
51–100 times	68 (9.84%)	47 (6.80%)
>100 times	67 (9.70%)	51 (7.38%)

**Weekly alcohol consumption**
No alcohol	191 (27.64%)
1–6 units	213 (30.82%)
7–12 units	123 (17.80%)
13–18 units	58 (8.39%)
19–24 units	38 (5.50%)
25–30 units	19 (2.75%)
31–36 units	17 (2.46%)
37–42 units	8 (1.16%)
43–48 units	7 (1.01%)
49–54 units	6 (0.89%)
55–60 units	4 (0.58%)
>60 units	7 (1.01%)

After providing these demographic data, subjects were given the opportunity to answer questions on up to four drug experiences: (1) their “most intense” psychedelic experience; (2) a “typical” psychedelic experience; (3) a “typical” cocaine experience; and (4) a “typical” alcohol experience. For psychedelic experiences subjects could further specify the drug taken (options were: LSD, psilocybin, mescaline, DMT and ayahuasca). For each experience subjects were asked to provide information on how long ago the experience was (options were: “Today”, “Last week”, “1–4 weeks” ago, “1–6 months” ago, “6–12 months” ago, “1–5 years” ago, “6–10 years” ago, and “Over 10 years” ago). They were also asked how “intense” the experience was (for psychedelic experiences), or how “energized/wired” or “inebriated/drunk” they felt (for cocaine and alcohol experiences, respectively) on a visual analog scale from 0 to 100, with 0 = “Not at all” and 100 = “The most intense/energized/inebriated imaginable”. The rationale for enquiring about a typical and most intense experience with psychedelics was to collect a greater range of possible responses with regards to psychedelics, which was our primary drug class of interest.

For each experience subjects were also asked to state the dose of the drug taken. For psychedelic drugs, subjects were asked to provide a “rough/ballpark” estimate using an LSD-equivalent dose; available options ranged from “No more than half a tab/50 micrograms of LSD” to “More than 3 tabs/300 micrograms of LSD”, split into 5 non-overlapping groups. This was done with the aim of providing a standard reference against which any non-LSD classical psychedelic could be compared. For cocaine, the dose options available ranged from: “less than 1/8 gram” to “More than 2 grams”, split into six non-overlapping groups. For alcohol, the dose options ranged from: “Less than 3 units” to “Over 24 units”, split into nine non-overlapping groups.

For each drug experience, subjects answered the question “Do you believe that the experience [induced by the relevant drug] and your contemplation of that experience have led to a change in your current sense of personal well-being or life satisfaction?” using a 7-point rating scale (+3 = “increased very much”; +2 = “increased moderately”; +1 = “increased slightly”; 0 = “no change”; −1 = “decreased slightly”; −2 = “decreased moderately”; and −3 = “decreased very much”), taken from the persisting effects questionnaire as per Barrett et al. (2015).

Subjects then answered the 16 ego-consciousness items (Table 1) relating to each specific drug experience in question. For experiences occasioned by psychedelic drugs, subjects additionally answered seven questions selected from the Mystical Experiences Questionnaire (MEQ; MacLean et al., 2012; Barrett et al., 2015). The seven questions selected have been shown to load onto a single common factor denoting “Mystical” experiences (Barrett et al., 2015), and all relate to the so-called “unitive” experience, which is considered to be a fundamental feature of the mystical experiences (Stace, 1960; Hood, 1975; James, 1985). The unitive experience is related to the notion of dissolved ego-boundaries, and it has been hypothesized (although never formally investigated) that the phenomenology of the unitive experience overlaps with that of ego-dissolution (James, 1882; Leary et al., 1964; Grof, 1980; Harrison, 2010). The inclusion of the relevant MEQ questions in this survey allowed us to explicitly test this hypothesis, and provided a means of measuring the convergent validity of the ego-dissolution construct.

The specific MEQ questions included, and their relevant MEQ30 identifiers (Barrett et al., 2015), were as follows: “Freedom from the limitations of your personal self and feeling a unity or bond with what was felt to be greater than your personal self” [MEQ30 Q14], “Experience of oneness in relation to an ‘inner world’ within” [MEQ30 Q20], “Experience of the fusion of your personal self into a larger whole” [MEQ30 Q26], “Experience of unity with ultimate reality” [MEQ30 Q28], “Feeling that you experienced eternity or infinity” [MEQ30 Q05], “Experience of oneness or unity with objects and/or persons perceived in your surroundings” [MEQ30 Q06] and “Experience of the insight that ‘all is One”’ [MEQ30 Q18]. Each item was rated on a 6-point scale, where 0 = “none, not at all”; 1 = “so slight cannot decide”; 2 = “slight”; 3 = “moderate”; 4 = “strong (equivalent in degree to any previous strong experience or expectation of this description)”; and 5 = “extreme (more than ever before in my life and stronger than four)”. This is consistent with the standard procedure for completion of the MEQ.

### Dissemination of the Survey

This study was approved by the local ethics committee. The survey was implemented and hosted by the online service Survey Gizmo^1^, and was estimated to take 38 min to complete. Survey Gizmo has comprehensive privacy policies and security features that maintain the anonymity of responses in line with ethics requirements.

Participants were recruited to take the online survey via web-link advertisements posted on Facebook groups, Twitter pages, email newsletters, and online drug forums with a short request (“please participate in our anonymous online questionnaire designed to learn more about experiences with classical psychedelics, cocaine, and alcohol”). Recruitment targeted online communities interested in psychoactive substances and altered states of consciousness (e.g., Psychedelic Society^2^, and Multidisciplinary Association for Psychedelic Studies^3^), as well as websites visited by more diverse populations, (e.g., Reddit^4^, and Mumsnet^5^). The collection of IP addresses and geographical locations of participants was disabled and participants were informed of the anonymity of their responses. After reading a summary of the inclusion criteria and instructions, participants provided informed consent by clicking “next” on the first page of the questionnaire.

Inclusion criteria for participants were: (1) at least 18 years of age; and (2) had had at least one experience with a classical psychedelic (LSD, psilocybin, DMT, ayahuasca or mescaline), cocaine, and/or alcohol. Data collection occurred over a 4-week period.

### Statistical Analysis

#### Factor Analysis and Definition of Ego-Dissolution Inventory

We defined a completed form as one in which the subject had answered all 16 ego-consciousness items relating to at least one drug experience, and also provided information about the dose of drug taken, the subjective intensity of the experience, and the effect on well-being.

Scores for the 16 ego-consciousness items for each complete form were subjected to an exploratory factor analysis using the iterated principle axis factor method and an oblique (promax) rotation, which allowed common factors to be correlated (Budaev, 2010). The appropriate number of factors to be extracted was determined by parallel analysis of principal components using 1000 random draws (Horn, 1965; O’Connor, 2000) and Cattell’s scree plot criterion (Cattell, 1966). Based on the factor loadings (from the pattern matrix), the 16 ego-consciousness items could be easily separated into two 8-item scales reflecting “ego-dissolution” and “ego-inflation” experiences (full details in “Results” Section). The mean item scores for both the ego-dissolution and ego-inflation scales were used as a measure of ego-dissolution and ego-inflation for all subsequent analysis.

#### Reliability and Construct Validity

Internal consistency of the scales was assessed with Cronbach’s alpha (Cronbach, 1951). Convergent validity of the ego-dissolution scale was assessed using the correlation with our MEQ-derived measure of unitive experience for psychedelic experiences. Discriminant validity was demonstrated firstly by the ability of an exploratory factor analysis to separate the eight ego-dissolution items from the eight ego-inflation items, and secondly by demonstrating the specificity of ego-dissolution for the psychedelic experience over alcohol or cocaine experiences (see “Relationship of Ego-Dissolution to Psychedelic Drugs and Persisting Effects” Section).

#### Relationship of Ego-Dissolution to Psychedelic Drugs and Persisting Effects

The specificity of the relationship between ego-dissolution and experiences occasioned by classical psychedelics was tested in three ways. Firstly we investigated the correlation between reported drug dose and both ego-dissolution and ego-inflation for each drug class separately (where drug dose was defined as the central value for the selected dose range for each experience). For each drug class the null hypothesis that the dose-ego-dissolution and dose-ego-inflation correlations were equal was tested using a 2-tailed *t*-test of the differences between dependent correlation coefficients (Field, 2013).

Secondly, we investigated the relationship between the reported subjective intensity of the experience and ego-dissolution. Correlations between experience intensity and ego-experiences within a drug class were investigated in an identical manner to the dose-response relationships. As ratings of subjective experience intensity are theoretically comparable across drug classes (unlike drug doses) we were able to test the hypothesis that the linear relationship between subjective intensity and ego-dissolution (or ego-inflation) was different between psychedelic, cocaine and alcohol experiences by testing whether the slope of the linear regression line relating subjective intensity (independent variable) to ego-dissolution or ego-inflation (dependent variables) is significantly different between drug classes (using MATLAB’s aoctool [Analysis of covariance (ANCOVA)] function [MATLAB 2015b, Mathworks]).

Finally, a Support Vector Machine (SVM) classifier (a common supervised machine learning algorithm) was trained to distinguish between typical experiences with psychedelics, cocaine and alcohol using only the ego-dissolution and ego-inflation score for each experience, in three binary one-v-one classification tasks (psychedelic vs. cocaine, psychedelics vs. alcohol, cocaine vs. alcohol). Only “typical” psychedelic experiences were included in this analysis to avoid classification problems associated with numerically imbalanced classes (He and Garcia, 2009). The SVM classifier was implemented in MATLAB 2015b (Mathworks), as part of the Classification Learner application, with the following settings: 5-fold cross-validation, linear kernel, standard normalization transformation applied to data before entering into SVM classifier.

The correlation between ego-dissolution or ego-inflation and reported changes in personal well-being was compared both between and within drug classes using Fisher’s *r*-to-*z* transform and 2-tailed *t*-test, respectively (Field, 2013).

Spearman’s rho was used to quantify all bivariate correlations. Statistical significance is defined as *p* < 0.05 (two-tailed). Multiple statistical comparisons were performed when analyzing the correlation between ego-dissolution or ego-inflation and variables of interest (e.g., drug dose, experience intensity, or change in well-being) separately for the three drug classes. In these instances, we applied Bonferroni correction for multiple comparisons (specifically, six simultaneous comparisons) such that differences are deemed statistically significant for *p* < 0.008. 95% confidence intervals were calculated using the bias corrected and accelerated bootstrap method (1000 samples). All statistical analysis was performed using SPSS Statistics (IBM, Version 22), and MATLAB (MathWorks, Version 2015b including Statistics and Machine Learning Toolbox).

## Results

### Baseline Demographics of Survey Responders

Six-hundred and ninety-one subjects completed the online survey. Table 2 summarizes the demographic information for these subjects. Each subject answered questions for a mean of 2.65 drug experiences (SD 1.18), providing data for 1828 complete drug experiences for analysis (1043 were with psychedelic drugs: 584 relating to the most intense psychedelic experience and 459 relating to a typical psychedelic experience, occurring a median of 1–5 years and 6–12 months prior to survey completion, respectively. 377 were with cocaine, occurring a median of 1–5 years prior to survey completion. 408 were with alcohol, occurring a median of 1–4 weeks prior to survey completion).

### Ego-Dissolution Inventory: Factor Structure and Internal Consistency

To investigate the factor structure of the 16 ego-consciousness items in a hypothesis-free manner, all questions were submitted to an exploratory factor analysis. The Kaiser–Meyer–Olkin measure of sampling adequacy was 0.918 and Bartlett’s test of sphericity was highly significant (χ^2^_(120)_ = 22441.7, *p* < 0.001) confirming that the data were indeed suitable for factor analysis (Budaev, 2010).

Both parallel analysis for principle components (Horn, 1965; O’Connor, 2000) and inspection of the scree plot using Cattell’s criterion (Cattell, 1966) supported a model with two factors or components (parallel analysis observed and 95% confidence interval simulated eigenvalues for the 3rd component were 1.04 and 1.12, respectively). The first component explained 36.6% of the variance in the sample, and the second component explained 29.5% of the variance in the sample. All other components explained <7% of the variance in the sample.

The data were therefore subjected to an exploratory factor analysis to extract two common factors. Factor 1 comprised of the eight items relating to the experience of “ego-dissolution” whilst Factor 2 comprised of the eight items relating to the experience of “ego-inflation”. Communality values (the proportion of an item’s variance that can be explained by the extracted common factors) ranged from 39% to 82%. Every item loaded strongly and exclusively onto either Factor 1 or Factor 2, demonstrating a simple and easily interpretable factor structure (Table 1).

Guided by the results of the exploratory factor analysis, two 8-item scales were derived, one reflecting the experience of “ego-dissolution”, and one reflecting the experience of “ego-inflation” (Table 1). Both scales had excellent internal consistency (Cronbach’s alpha = 0.93 and 0.91, respectively; Cronbach, 1951). The mean item scores for both the ego-dissolution and ego-inflation scale were almost perfectly correlated with the factor scores calculated using the regression method from the exploratory factor analysis output (both rho > 0.99, *p* < 0.001). For ease of interpretability and replication, the mean item scores from the two scales were used as a measure of ego-dissolution and ego-inflation for all subsequent analysis.

### Construct Validity

For experiences with psychedelic drugs, the score for the MEQ-derived measure of unitive experience correlated strongly with ego-dissolution (rho = 0.735 [95% CI 0.704, 0.763], *p* < 0.001) providing a clear demonstration of convergent validity. This measure of unitive experience also correlated positively with ego-inflation (rho = 0.274 [0.219, 0.332], *p* < 0.001) but the strength of this correlation was significantly weaker than that between unitive experience and ego-dissolution (*t*_(1040)_ = 17.8, *p* < 0.001). In the exploratory factor analysis, Factor 1 (“ego-dissolution”) and Factor 2 (“ego-inflation”) were essentially orthogonal (rho = −0.11), demonstrating the discriminant validity of ego-dissolution and ego-inflation subscales.

### Specificity of Ego-Dissolution for Psychedelic Drugs

#### Dose-Response Relationship

**Figure 1:**
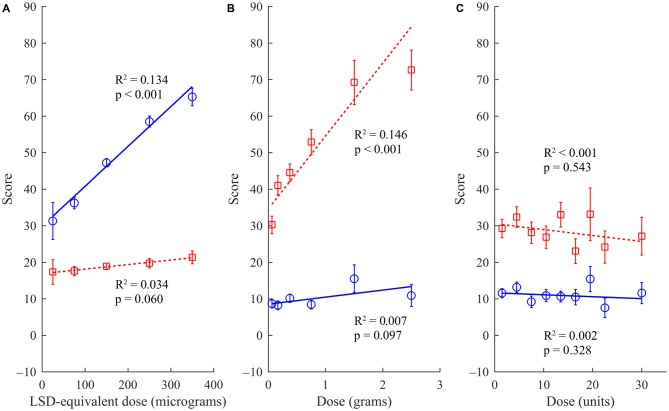
**Relationship between drug dose and scores for ego-dissolution (blue circles, solid line) and ego-inflation (red squares, dashed line) for experiences occasioned by different drug classes. (A)** Psychedelic experiences (*n* = 1043). **(B)** Cocaine experiences (*n* = 377). **(C)** Alcohol experiences (*n* = 408). Lines represent linear regression lines of best fit, with corresponding *R*^2^ and *p*-value. Error bars represent ±1 SEM.

For experiences with psychedelic drugs, there was a significant positive correlation between reported drug dose and ego-dissolution (rho = 0.371 [0.317, 0.427], *p* < 0.001), and only a weak correlation between reported drug dose and ego-inflation (rho = 0.063 [0.003, 0.127], *p* = 0.043), which did not survive correction for multiple comparisons (Figure 1A). The difference between these two correlations was significant (*t*_(1040)_ = 8.55, *p* < 0.001). Conversely, for cocaine, there was a significant and strong positive correlation between reported drug dose and ego-inflation (rho = 0.385 [0.390, 0.477], *p* < 0.001) but only a weak correlation between drug dose and ego-dissolution (rho = 0.115 [0.012, 0.211], *p* = 0.026), which also did not survive correction for multiple comparisons (Figure 1B). Again, the difference between these two correlations was significant (*t*_(374)_ = 4.88, *p* < 0.001). For alcohol there was no dose-response relationship with either ego-dissolution (rho = −0.055 [−0.150, 0.048], *p* = 0.266) or ego-inflation (rho = −0.054 [−0.148, 0.037], *p* = 0.328), and no difference between these correlations (*t*_(405)_ = 0.031, *p* = 0.488; Figure 1C).

#### Intensity-Response Relationship

For experiences with psychedelic drugs, the subjective intensity of the experience was positively correlated with both ego-dissolution (rho = 0.577 [0.529, 0.621], *p* < 0.001) and ego-inflation (rho = 0.099 [0.040, 0.159], *p* = 0.001), although the correlation with ego-dissolution was significantly stronger than that with ego-inflation (*t*_(1040)_ = 15.1, *p* < 0.001). The opposite pattern was observed for cocaine and alcohol experiences however, where there was a significantly stronger correlation between the subjective intensity of these drug experiences and ego-inflation (Cocaine: rho = 0.545 [0.464, 0.625], *p* < 0.001. Alcohol: rho = 0.502 [0.410, 0.582], *p* < 0.001) than ego-dissolution (Cocaine: rho = 0.279 [0.180, 0.378], *p* < 0.001. Alcohol: rho = 0.334 [0.237, 0.421], *p* < 0.001); (*t*_(374)_ = 5.33, *p* < 0.001, and *t*_(405)_ = 4.03, *p* < 0.001, for cocaine and alcohol respectively).

**Figure 2:**
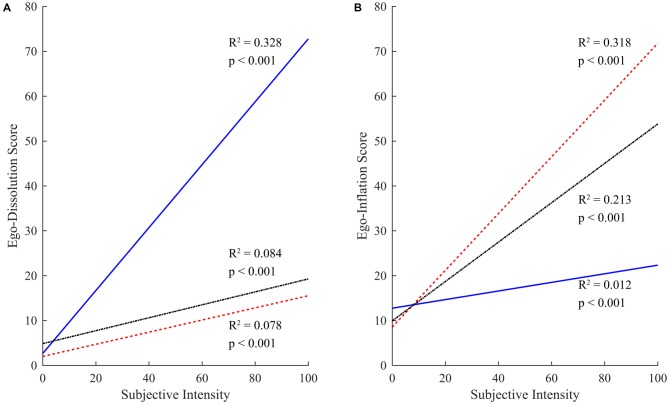
**Relationship between experience intensity and ego-consciousness. (A)** Linear regression lines of best fit for relationship between reported subjective intensity and ego-dissolution for experiences occasioned by psychedelic drugs (blue solid line, *R*^2^ = 0.328, *p* < 0.001), cocaine (red coarsely-broken line, *R*^2^ = 0.078, *p* < 0.001) or alcohol (black finely-broken line, *R*^2^ = 0.084, *p* < 0.001). **(B)** Linear regression lines of best fit for relationship between reported subjective intensity and ego-inflation for experiences occasioned by psychedelic drugs (blue solid line, *R*^2^ = 0.012, *p* < 0.001), cocaine (red coarsely-broken line, *R*^2^ = 0.318, *p* < 0.001) or alcohol (black finely-broken line, *R*^2^ = 0.213, *p* < 0.001).

ANCOVA analysis was used to fit separate regression lines relating subjective intensity (predictor variable) to ego-dissolution or ego-inflation (dependent variables), for each drug class separately. This analysis confirmed that ego-dissolution experiences were significantly predicted by experience intensity (*F*_(1,1822)_ = 528.4, MSE = 165132.7, *p* < 0.001), drug class (*F*_(2,1822)_ = 636.39, MSE = 198863.7, *p* < 0.001), and the interaction between subjective intensity and drug class (*F*_(2,1822)_ = 116.29, MSE = 36338, *p* < 0.001). Follow-up multiple comparison tests (Tukey’s HSD criterion) demonstrated that the slope of the regression line relating experience intensity to ego-dissolution was significantly steeper for psychedelics (unstandardized regression coefficient = 0.701 [0.640, 0.762]), than for cocaine (0.135 [0.088, 0.182]) or alcohol (0.144 [0.098, 0.191], both *p* < 0.001). There was no difference between the slopes of the regression lines relating experience intensity to ego-dissolution for alcohol vs. cocaine (*p* = 0.984; Figure 2A).

Ego-inflation experiences were also significantly predicted by subjective intensity (*F*_(1,1822)_ = 229.16, MSE = 89120.9, *p* < 0.001), drug class (*F*_(2,1822)_ = 278.5, MSE = 108309.5, *p* < 0.001) and the interaction between drug class and intensity (*F*_(2,1822)_ = 61.16, MSE = 23785.8, *p* < 0.001). The slope of the regression line relating experience intensity to ego-inflation, was, however, significantly shallower for psychedelics (0.096 [0.043, 0.149]) compared with cocaine (0.632 [0.538, 0.726], *p* < 0.001) and alcohol (0.439 [0.357, 0.521], *p* < 0.001), and was steeper for cocaine than for alcohol experiences (*p* = 0.003; Figure 2B).

#### Support Vector Machine Classifier

**Figure 3:**
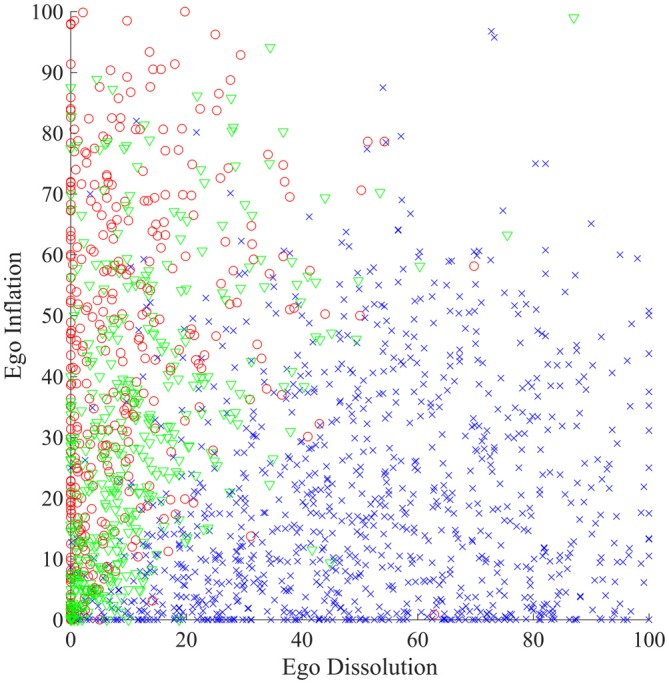
**The relationship between ego-dissolution and ego-inflation for experiences occasioned by classical psychedelics (blue crosses), cocaine (red circles) and alcohol (green triangles)**.

As a final demonstration of the specificity of the ego-dissolution experience for psychedelic drugs, we trained a SVM binary classifier to distinguish between typical psychedelic (*n* = 459), cocaine (*n* = 377) and alcohol (*n* = 408) drug experiences using only the ego-dissolution and ego-inflation scores. This classifier achieved an accuracy of 90.1% when distinguishing between psychedelic vs. cocaine experiences (receiver-operator characteristic (ROC) area under the curve (AUC) = 0.958), and 85.2% accuracy when distinguishing between psychedelic and alcohol experiences (ROC AUC = 0.927). By contrast, it performed poorly when distinguishing cocaine from alcohol experiences (63.4% accuracy, ROC AUC = 0.685). Classification at chance level is equivalent to 50% accuracy and ROC AUC = 0.5. Figure 3 illustrates the relationship between ego-dissolution and ego-inflation for experiences occasioned by different drugs.

### Ego-Dissolution and Well-Being

As a final exploratory analysis, we sought to investigate the relationship between ego-experiences and the extent to which the experience in question changed subjects’ current sense of personal well-being or life satisfaction. For psychedelic drugs, the median response on the 7-point rating scale (with possible answers from −3 to +3) was +2 (+2 = “Increased moderately”, interquartile range = 2, skewness = −0.769, median time elapsed since experience = 1–5 years), which was significantly greater than the median response for cocaine (0 = “No change”, interquartile range = 0, skewness −0.135, median time elapsed since experience = 1–5 years) or alcohol (0 = “No change”, interquartile range = 0, skewness = −0.220, median time elapsed since experience = 1–4 weeks; *p* < 0.001 for both comparisons, Wilcoxon rank sum test). The difference between the mean change in well-being between cocaine and alcohol was not significant (*p* = 0.260, Wilcoxon rank sum test).

There was a trend for a positive correlation between ego-dissolution score and improvement in well-being across all drug classes, with this effect reaching statistical significance after correction for multiple comparisons only for experiences occasioned by psychedelic drugs (psychedelic: rho = 0.392 [0.342, 0.442], *p* < 0.001, cocaine: rho = 0.103 [−0.006, 0.204], *p* = 0.045; alcohol: rho = 0.084 [−0.009, 0.181], *p* = 0.091). This correlation was significantly stronger for psychedelic drugs compared with either cocaine (*Z* = 5.15, *p* < 0.001) or alcohol (*Z* = 5.63, *p* < 0.001), but did not differ between cocaine and alcohol drug experiences (*Z* = 0.267, *p* = 0.789).

Ego-inflation was positively correlated with improvement in well-being for psychedelic drugs (rho = 0.198 [0.135, 0.263], *p* < 0.001). There was a trend for a negative correlation between ego-inflation and well-being for experiences with cocaine and alcohol however, although these correlations were non-significant after correction for multiple comparisons (Cocaine: rho = −0.083 [−0.198, 0.027], *p* = 0.107. Alcohol: rho = −0.112 [−0.216, −0.014], *p* = 0.024). Whilst the correlation between ego-inflation and well-being was significantly different for psychedelic drugs compared with both cocaine (*Z* = 4.71, *p* < 0.001) and alcohol (*Z* = 5.53, *p* < 0.001), it was not significantly different between cocaine and alcohol (*Z* = 0.408, *p* = 0.683).

For psychedelic experiences, the positive correlation between ego-dissolution and increase in well-being was stronger than between ego-inflation and change in well-being (*t*_(1040)_ = 5.48, *p* < 0.001). For both cocaine and alcohol experiences, the negative correlation between ego-inflation and change in well-being was stronger than the correlation between ego-dissolution and change in well-being (Cocaine: *t*_(374)_ = 3.14, *p* = 0.001. Alcohol: *t*_(405)_ = 4.13, *p* < 0.001).

## Discussion

The results presented in this study demonstrate the internal consistency, single-factor psychometric structure and construct validity the EDI, a new 8-item self-report questionnaire designed to measure the experience of ego-dissolution. Additionally, our results demonstrate that ego-dissolution positively correlates with drug dose and experience intensity specifically for psychedelic drugs, compared with cocaine or alcohol experiences. This result mirrors the previously demonstrated positive relationship between psilocybin dose and altered states of consciousness, including “oceanic boundlessness” and “dread of ego dissolution” (as measured by the 5D-ASC questionnaire; Hasler et al., 2004; Wittmann et al., 2007; Studerus et al., 2011) and the mystical experience (Griffiths et al., 2011). Indeed, we also found a positive correlation between psychedelic dose and unitive experience (rho = 0.307, *p* < 0.001).

The experience of a coherent and well-circumscribed self is a cardinal feature of adult human waking consciousness (Carhart-Harris and Friston, 2010; Carhart-Harris et al., 2014). Conversely, the experience of ego-dissolution is unfamiliar to most people, and is related to relatively rare altered states of consciousness, such as the psychedelic experience (Huxley, 1954; Leary et al., 1964; Grof, 1980; Harrison, 2010; Carhart-Harris et al., 2014; Lebedev et al., 2015) and the mystical experience (Stace, 1960; James, 1985). Despite the relative rarity of the ego-dissolution experience, a fuller understanding of its neurobiological correlates may inform our understanding of the therapeutic mechanism of action of psychedelic drugs (Grof, 1980; Griffiths et al., 2008, 2011) and human consciousness more generally (Carhart-Harris et al., 2014). The development and validation of the EDI is an important contribution to this research programme.

An understanding of the neurobiological correlates of self-experience is also of great importance to a number of mental health conditions, where the sense of self is disrupted or compromised (Northoff, 2014). A disrupted sense of self has long been considered to be a core phenomenological feature of acute psychosis (Federn, 1952; Laing, 1959; Fischman, 1983; Jaspers, 1997; Scharfetter, 1981; Nour and Barrera, 2015). Recently this idea has been extended by Sass and Parnas (2003), who propose that the core phenomenological alteration in schizophrenia is an *“instability of pre-reflective self-awareness”*, which has been termed an “ipseity-disorder” or “self-disorder” (Sass et al., 2011). They argue that saturating all subjective experience is a pre-reflective awareness of self as the unified subject of experience (Sass and Parnas, 2003). This notion is closely related to the feeling of inhabiting a living body embedded in the world (Stanghellini, 2009). As the pre-reflective sense of self is related to a feeling of immersion in a social world, ipseity disturbance may also result in the deficits in social cognition seen in patients (Parnas and Bovet, 1991; Stanghellini, 2001, 2009; Nelson et al., 2009; Nordgaard and Parnas, 2014; Nour and Barrera, 2015). Further research is required to clarify the relationship between the self-disturbances seen in schizophrenia and psychedelic states, as well as the relationship between other features of the psychedelic state and psychosis (Corlett et al., 2009).

Our results represent a necessary step in the demonstration of the construct validity of ego-dissolution. Construct validity can be decomposed into discriminant and convergent validity. Discriminant validity of the EDI was demonstrated by the fact that items relating to ego-dissolution and those relating to ego-inflation loaded onto two orthogonal factors in the exploratory factor analysis. Convergent validity of the EDI was demonstrated by the strong positive correlation between EDI and our (MEQ-based) measure of the unitive experience. This suggests that experiences of ego-dissolution, unity and dissolved ego-boundaries may be conceptually inseparable (Federn, 1952; Savage, 1955; Fischman, 1983), occurring together during “peak” psychedelic experiences. Consistent with this hypothesis, the item “I felt at one with the universe” loaded particularly strongly on the “ego-dissolution” factor (0.830), together with items explicitly referring to “dissolution” and “disintegration” of self or ego (0.883 and 0.897, respectively).

Our measure of ego-inflation, in contrast to ego-dissolution, showed a significant dose-response relationship with cocaine, but not psychedelics or alcohol. Although all three drug classes showed a positive correlation between ego-inflation and experience intensity, this relationship was strongest for cocaine and weakest for psychedelic drugs. These results suggest that experiences occasioned by cocaine are in some sense antithetical to the psychedelic experience; with cocaine seeming to promote self-centeredness rather than the selflessness associated with psychedelics.

Consistent with this hypothesis, a binary SVM classifier was able to identify experiences occasioned by psychedelic drugs vs. cocaine or alcohol with over 85% accuracy using the ego-dissolution and ego-inflation scores alone. This machine-learning approach may be usefully applied in a number of contexts. For example, it may help to inform debates over whether hybrid compounds such as 3,4-methylenedioxymethamphetamine (MDMA) should be classed as “ego-dissolving” psychedelic-like agents, “ego-inflating” stimulant-like agents, both, or neither. Moreover, used in conjunction with neurobiological measures, such as neuroimaging, these tools may help us to identify key (defining) properties of different psychoactive drugs, as well as non-drug-induced states, in a data-driven manner, and may inform novel hypotheses concerning the endogenous role of 5-HT_2A_ receptors. This may help us to address questions regarding the similarities and differences of different altered states of consciousness such as dreaming, psychosis and the psychedelic state (Carhart-Harris, 2007). More generally, our results suggest that the specific way in which a drug disturbs ego-consciousness may inform a novel phenomenologically-based classification system for psychoactive substances.

Subjects in the present study reported that on average their reported experiences with psychedelic drugs had a positive and lasting impact on their well-being, which correlated positively with the degree of ego-dissolution experienced (rho = 0.392). This is consistent with previous work, which has established that mystical (or “peak”) experiences occasioned by psilocybin correlate positively with increases in “openness” (MacLean et al., 2011), well-being (Barrett et al., 2015), and the meaningfulness/spiritual significance of the experience (Griffiths et al., 2008). Similarly, one influential model of LSD therapy states that the experience of “ego death and … loss of boundaries between the subject and the objective world, with ensuing feelings of unity” is of great therapeutic benefit (Grof, 1980). Moreover, lifetime psychedelic use has been associated with reduced odds ratio of psychological distress, suicidality and certain mental health problems in large population samples (Krebs and Johansen, 2013; Hendricks et al., 2015; Johansen and Krebs, 2015).

Regarding the neurobiology of the psychedelic state, previous work has indicated that psychedelics disrupt the integrity of the default-mode network (DMN), a normally well-integrated network of (mostly cortical) brain regions that display high metabolic demands, “connector-hub” status and appear to be involved in high-level functions such as the processing of self-specific information (Qin and Northoff, 2011; Buckner, 2013; Speth et al., 2016). Psilocybin and DMT-containing ayahuasca decrease functional connectivity between key DMN hub regions (Carhart-Harris et al., 2012; Muthukumaraswamy et al., 2013; Palhano-Fontes et al., 2015). Psilocybin and LSD also disrupt the functional segregation between usually well-demarcated brain networks, promoting increased global integration (Carhart-Harris et al., 2013; Roseman et al., 2014), which correlates with ego-dissolution (Tagliazucchi et al., 2016). Disrupted integrity of the DMN and reduced anticorrelation between DMN and task-positive network activity may facilitate a less constrained style of cognition and a weakening of the feeling of a well-circumscribed self. This may be a psychological consequence of a less constrained (more entropic) style of brain activity and a “collapse” in the normal hierarchical organization of cortical circuits, which normally functions to finesse perception and cognition by minimizing uncertainty (Hohwy, 2007; Carhart-Harris and Friston, 2010; Friston, 2010; Carhart-Harris et al., 2014; Roseman et al., 2014; Tagliazucchi et al., 2014; Nour and Nour, 2015).

These previous experimental findings suggest that the integrity of the DMN may be important for normal self-experience (Qin and Northoff, 2011; Carhart-Harris et al., 2014). Relatively few studies have explicitly investigated the neural correlates of ego-dissolution experiences occasioned by psychedelic drugs. Muthukumaraswamy et al. (2013) found that the experience of ego-disintegration occasioned by psilocybin correlated with decreased alpha power in the posterior cingulate cortex, a DMN hub region, using magnetoencephalography (MEG). Lebedev et al. (2015) by contrast, found that ego-dissolution correlated with decreased functional connectivity between the anterior parahippocampal cortex and higher-level cortical DMN regions as well as decreased (within-network) integrity of the salience network, and reduced inter-hemispheric communication.

A recent study found that the degree of ego-dissolution occasioned by LSD was correlated with global functional connectivity (“Functional Connectivity Density”, as measured by functional magnetic resonance imaging (fMRI)) in bilateral temporo-parietal junction (angular gyrus) and bilateral insular cortex (Tagliazucchi et al., 2016). More generally, this study reported that LSD induced increases in global connectivity in more widespread high-level association cortices, which overlap substantially with the default mode network. A separate analysis of the same fMRI data further revealed that ego-dissolution showed a strong inverse correlation with DMN network integrity (measured as within-network resting state functional connectivity), and functional connectivity between the parahippocampus and retrosplenial cortex (Carhart-Harris et al., 2016). Moreover, significant relationships were found between ego-dissolution and decreased delta and alpha power (e.g., in posterior cingulate cortex) as measured by MEG (Carhart-Harris et al., 2016), replicating previous findings with psilocybin (Muthukumaraswamy et al., 2013).

One limitation of these studies is that they either used a single-item measure of ego-dissolution (Muthukumaraswamy et al., 2013; Carhart-Harris et al., 2016; Tagliazucchi et al., 2016), or principle component analysis loadings from an (as yet) unvalidated questionnaire which included items indirectly related to ego-dissolution (e.g., perceptual abnormalities; Lebedev et al., 2015). Thus, the validated EDI allows for a more rigorous study of the neural correlates of ego-dissolution experienced in a number of altered states of consciousness, including those associated with psychedelic drugs and spiritual practice.

The present study has some limitations. Firstly, the population sampled was fairly homogenous, which limits the study’s external validity, and thus our ability to extrapolate to a broader demographic. Specifically, most of our subjects were male, under the age of 30, and had at least some university education. Over half the subjects had used classical psychedelic drugs on over 10 occasions. This also raises the possibility that our subjects’ responses were biased by their familiarity with reports about paradigmatic features of the psychedelic experience, such as ego-dissolution. Moreover, we did not collect information on the setting in which the psychedelic experiences took place, which is known to influence the quality of the experience (Leary et al., 1964; Grof, 1976; Fischman, 1983). Another important limitation of our study is its retrospective design, which introduces potential inaccuracies in experience recall. We employed an anonymous internet questionnaire design to facilitate the collection of a large data sample of subjects from around the world. Although this approach has its strengths, it is impossible to verify that the experiences attributed to psychedelics were indeed caused by these substances. Finally, we asked subjects to estimate the dose of psychedelic ingested using “LSD-equivalent” doses, so as to more easily investigate dose-response relationships across different classical psychedelics. The inaccuracy introduced by this approach, however, is likely to have weakened, rather than strengthened, any observed dose-response relationship between psychedelics and ego-dissolution.

Given these limitations, future studies should include a more heterogeneous sampling population, perhaps by intentionally recruiting subjects from different cultural and religious backgrounds, to explore the influence of these factors on ego-dissolution. It would also be of interest to investigate the relationship between responses on the EDI and other validated scales, for example the 5D-ASC (Studerus et al., 2010). Finally, experimental studies in which a range of psychedelic drug doses are administered to subjects in a blinded manner would be able to assess how subject-specific factors influence the relationship between dose and subjective effects, whilst controlling for recall effects and the effect of setting.

In conclusion, the present study offers initial-phase validation of the EDI, and adds to the growing evidence that ego-dissolution is a key phenomenological feature of the psychedelic experience, which may be studied experimentally. The existence of the EDI will facilitate future research into the neural correlates of this experience, which is of relevance for psychedelic-assisted psychotherapy and the phenomenology of certain psychiatric conditions.

## Author Contributions

MMN and RLC-H conceived of and designed this study and interpreted the results; contributed to drafting the work and revised it critically for important intellectual content. All authors approved the final version of this manuscript to be published and agreed to be accountable for all aspects of the work in ensuring that questions related to the accuracy or integrity of any part of the work be appropriately investigated and resolved. MMN and LE undertook statistical analysis of the data. LE managed the implementation of the questionnaire and subsequent data collection. MMN wrote the article, with editing from RLC-H.

## Funding

MMN is funded by the Medical Research Council, UK. RLC-H is funded by Mosley Foundation. DN is funded by Safra Foundation.

## Conflict of Interest Statement

The authors declare that the research was conducted in the absence of any commercial or financial relationships that could be construed as a potential conflict of interest.

## Footnotes

^1^http://www.surveygizmo.eu/

^2^http://www.psychedelicsociety.org.uk

^3^http://www.maps.org

^4^https://www.reddit.com/

^5^http://www.mumsnet.com/
